# Nodular Regenerative Hyperplasia Suggested by Noninvasive Findings during Adjuvant Trastuzumab Emtansine Therapy for Human Epidermal Growth Factor Receptor 2-Positive Breast Cancer

**DOI:** 10.70352/scrj.cr.26-0344

**Published:** 2026-09-17

**Authors:** Hideyuki Yamada, Rikiya Nakamura, Shouko Hayama, Reina Okuya, Keiko Oshida, Masaki Oshida, Hideyuki Hashimoto, Yasushi Ito, Yoshito Ota, Atushi Yoshii, Takahito Masuda, Akinobu Araki, Kentaro Sudo

**Affiliations:** 1 Chiba Cancer Center, Chiba, Chiba, Japan; 2 Cosmos Clinic, Ichihara, Chiba, Japan; 3 Chiba Foundation for Health Promotion & Disease Prevention, Chiba, Chiba, Japan; 4 Ito Shinkemigawa Clinic, Chiba, Chiba, Japan; 5 Department of Surgery, Sanmu Medical Center, Chiba, Chiba, Japan; 6 Tokyo City Clinic Ryogoku, Tokyo, Japan; 7 Masuda Clinic, Funabashi, Chiba, Japan

**Keywords:** breast cancer, HER2-positive, trastuzumab emtansine, nodular regenerative hyperplasia, portal hypertension, case report

## Abstract

**INTRODUCTION:**

Trastuzumab emtansine (T-DM1) is the standard adjuvant therapy for patients with human epidermal growth factor receptor 2 (HER2)-positive breast cancer with residual invasive disease after neoadjuvant chemotherapy. It is generally well tolerated. However, nodular regenerative hyperplasia (NRH), presenting as newly detected hepatic nodules, is a rare hepatic adverse event of T-DM1 that requires differentiation from liver metastasis. Here, we report a patient with HER2-positive breast cancer who developed new hepatic nodules during adjuvant T-DM1 therapy; NRH was suspected based on noninvasive findings.

**CASE PRESENTATION:**

A 58-year-old woman was referred for the evaluation of a right breast mass. Her past medical history was notable only for first-degree atrioventricular block, and her mother had a history of colon cancer. Imaging showed a 3.2-cm mass, and core needle biopsy revealed an invasive ductal carcinoma that was hormone receptor-positive and HER2-positive; the clinical stage was cT2N0M0 (stage IIA). She received neoadjuvant dose-dense doxorubicin/cyclophosphamide, followed by docetaxel plus trastuzumab and pertuzumab, and then underwent breast-conserving surgery with a sentinel lymph node biopsy. Residual invasive disease was identified (pathological complete response was not achieved). After whole-breast irradiation, T-DM1 and an aromatase inhibitor were administered as adjuvant therapy. After T-DM1 initiation, grade 1 transaminase elevation and grade 1 or 2 thrombocytopenia developed. Following 9 cycles of T-DM1, gadolinium-ethoxybenzyl-diethylenetriamine pentaacetic acid-enhanced MRI demonstrated new nodules in hepatic segments III and IV. Various hepatic lesions, including liver metastases and primary hepatic tumors, were considered in the differential diagnosis, but the imaging findings were atypical. T-DM1-associated NRH was also considered; however, at the patient’s request, a liver biopsy was not performed, and the lesions were followed up with imaging. The patient completed 14 cycles of T-DM1. Post-treatment imaging revealed mild splenomegaly and a coarse hepatic parenchymal texture, suggestive of mild portal hypertension, while the hepatic nodules remained stable. One year after the completion of T-DM1, her liver enzyme levels and platelet counts improved, and there was no evidence of breast cancer recurrence.

**CONCLUSIONS:**

When new hepatic nodules develop during adjuvant T-DM1 therapy, T-DM1-associated NRH should be considered in the differential diagnosis. Although histological confirmation is required for a definitive diagnosis, noninvasive findings such as thrombocytopenia, splenomegaly, atypical EOB-MRI findings, and lesion stability may raise suspicion of NRH and help distinguish it from liver metastases.

## Abbreviations

ALT
: alanine aminotransferase
AST
: aspartate aminotransferase
DWI
: diffusion-weighted imaging
EOB
: gadolinium-ethoxybenzyl-diethylenetriamine pentaacetic acid-enhanced
HER2
: human epidermal growth factor receptor 2
NRH
: nodular regenerative hyperplasia
T-DM1
: trastuzumab emtansine

## INTRODUCTION

T-DM1, an antibody–drug conjugate in which trastuzumab is conjugated to the microtubule inhibitor DM1 (a derivative of maytansine 1) via a linker, is an effective therapeutic option for HER2-positive breast cancer.^1)^ The KATHERINE trial established T-DM1 as the standard adjuvant therapy for HER2-positive breast cancer with residual invasive disease following neoadjuvant chemotherapy.^2)^ However, T-DM1 is associated with adverse events such as hepatotoxicity and thrombocytopenia, and rare cases of NRH have been reported in clinical trials and real-world practice.^1,2)^

When new hepatic nodules are detected during adjuvant T-DM1 therapy, distinguishing drug-related NRH from liver metastasis is clinically important because management differs substantially. Few reports have described early suspicion of NRH during T-DM1 therapy based on imaging and clinical findings. Here, we report a patient with HER2-positive breast cancer who developed new hepatic nodules during adjuvant T-DM1 therapy. Although liver metastasis was initially a major concern, the clinical course and imaging findings raised suspicion of T-DM1-associated NRH.

## CASE PRESENTATION

A 58-year-old woman was referred to our institution for assessment of a right breast mass. Her past medical history was notable only for first-degree atrioventricular block, and she was not taking regular medications. Her mother had a history of colon cancer. The initial laboratory test results were within normal limits, including those for liver enzymes (aspartate aminotransferase [AST] 17 U/L; alanine aminotransferase [ALT] 14 U/L). Mammography showed a spiculated mass in the upper outer quadrant of the right breast (**Supplementary Fig. 1**). Breast ultrasonography revealed a corresponding 3.2-cm hypoechoic mass and a mildly enlarged axillary lymph node (**Supplementary Fig. 2A** and **2B**). Core needle biopsy revealed invasive ductal carcinoma (histological grade 2), with estrogen receptor positivity in 90% of the tumor cells, progesterone receptor positivity in 10%, and a HER2 immunohistochemistry score of 3+. Fine-needle aspiration cytology of the axillary lymph node was negative for malignancy. Contrast-enhanced CT revealed no evidence of distant metastasis or hepatic lesions; however, a pancreatic cyst was identified. Contrast-enhanced breast MRI demonstrated a 3.2-cm enhancing mass. The clinical stage was cT2N0M0 (stage IIA).

Neoadjuvant chemotherapy was administered with dose-dense doxorubicin/cyclophosphamide for 4 cycles, followed by docetaxel plus trastuzumab and pertuzumab for 4 cycles. A clinical partial response was achieved. The patient subsequently underwent breast-conserving surgery with a sentinel lymph node biopsy. Pathological assessment revealed residual invasive ductal carcinoma measuring 15 mm (histological grade 3) without lymphatic or vascular invasion. Immunohistochemistry revealed estrogen and progesterone receptor positivity (90% and 60%, respectively), with HER2 expression scored as 1+ and a Ki-67 index of 70%. The pathological response to neoadjuvant therapy was grade 1b, and a pathological complete response was not achieved.

**Fig. 1:**
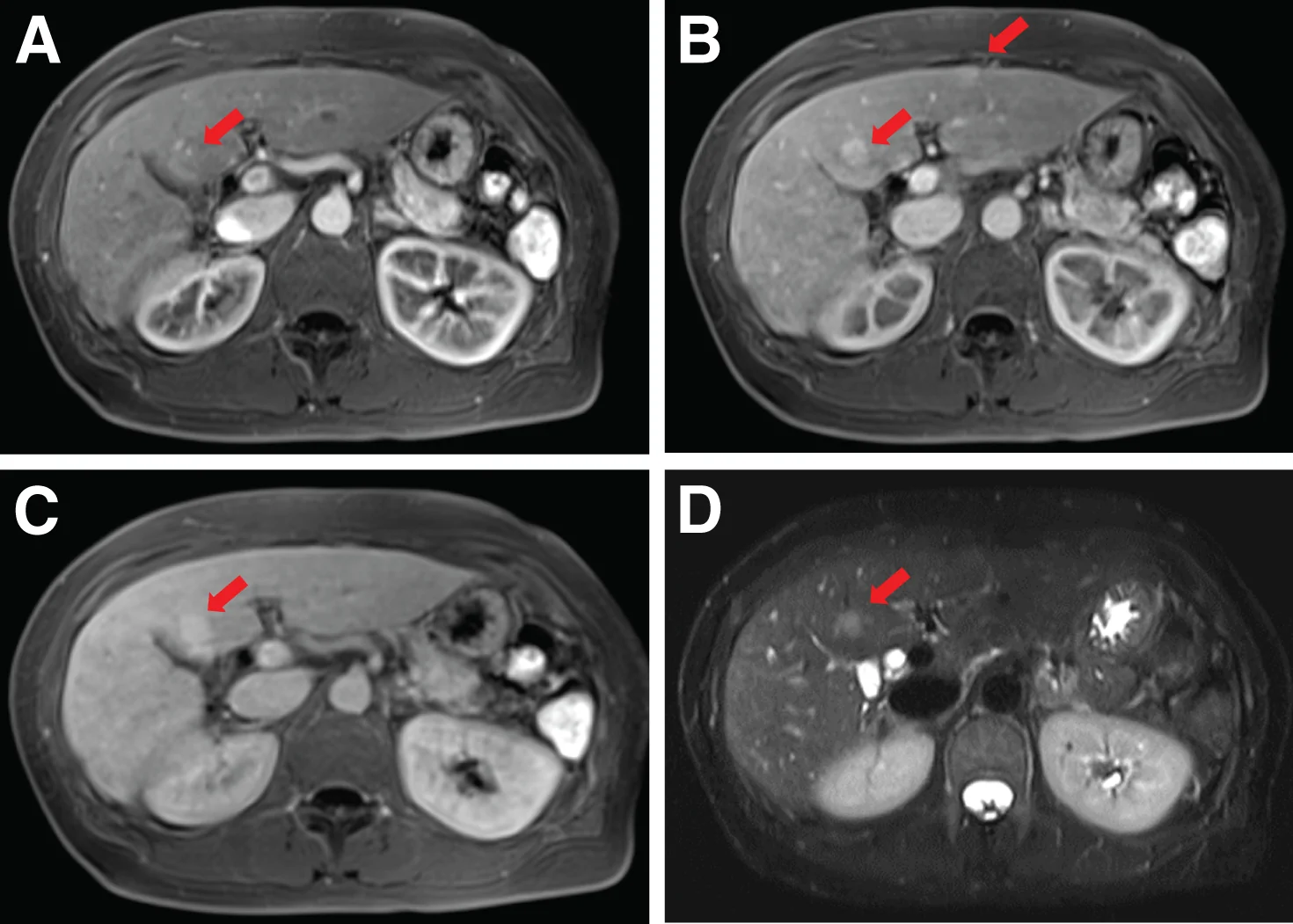
EOB-MRI showing hepatic lesions in segments IV and III during T-DM1 therapy. (**A**) Arterial phase image showing faint arterial phase hyperenhancement of the lesions. (**B**) Portal venous phase and (**C**) transitional phase images showing persistent hyperintensity. (**D**) Hepatobiliary phase image showing mild hyperintensity of the segment III lesion and approximately isointensity of the segment IV lesion, without definite hepatobiliary phase defects. EOB, gadolinium-ethoxybenzyl-diethylenetriamine pentaacetic acid-enhanced; T-DM1, trastuzumab emtansine

After whole-breast irradiation, adjuvant T-DM1 was initiated and planned for 14 cycles, with concomitant aromatase inhibitor therapy. Liver function was normal at the initiation of T-DM1. Mild AST elevation and grade 1 thrombocytopenia were observed after the first cycle (AST, 35 U/L; ALT, 29 U/L; platelet count, 119000/µL). After 9 cycles of T-DM1, abdominal MRI performed for follow-up of a pancreatic cyst revealed newly detected hepatic nodules. A DWI-hyperintense lesion was observed in liver segment IV (**Supplementary Fig. 3**). Abdominal ultrasonography also demonstrated corresponding hypoechoic lesions in hepatic segments IV and III (**Supplementary Fig. 4**). Subsequent EOB-MRI demonstrated 3 nodular lesions located in segment IV, the anterior aspect of segments III/IV, and the anterior aspect of segment III, including dominant lesions measuring 13 mm in segment IV and 14 mm in segment III (**Fig. 1**). These lesions had not been identified on pretreatment contrast-enhanced CT. The lesions showed mild hyperintensity on T2-weighted imaging and hyperintensity on DWI. Dynamic contrast-enhanced imaging demonstrated arterial phase hyperenhancement, with persistent hyperintensity during the portal venous and transitional phases. In the hepatobiliary phase, the segment III lesion showed mild hyperintensity, whereas the segment IV lesion was approximately isointense, without a definite hepatobiliary phase defect. Although liver metastasis and hepatocellular carcinoma were considered in the differential diagnosis because the lesions were newly detected, the imaging findings were atypical, and serum levels of alpha-fetoprotein and protein induced by vitamin K absence or antagonist-II were not elevated.

**Fig. 2:**
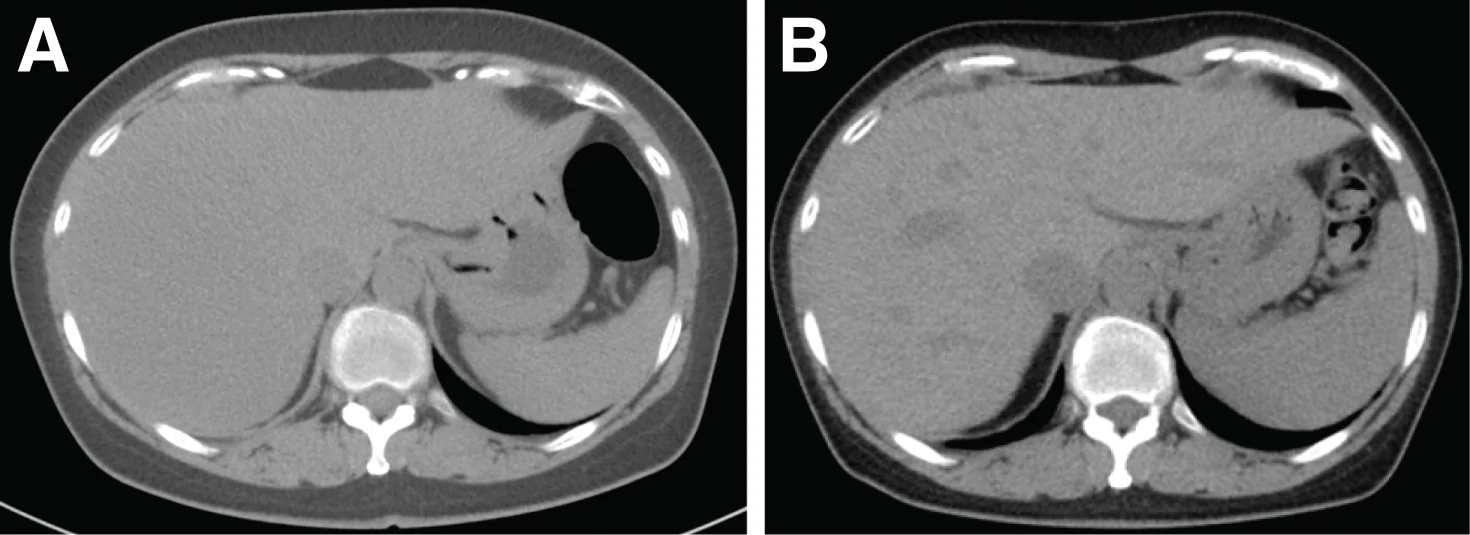
Non-contrast CT images before and after T-DM1 therapy. (**A**) Before initiation of T-DM1 therapy, the spleen measured 95.6 × 35.1 mm. (**B**) Two months after completion of T-DM1 therapy, the spleen had enlarged to 111.9 × 46.1 mm, consistent with splenomegaly. T-DM1, trastuzumab emtansine

After 13 cycles of T-DM1, EOB-MRI showed no marked changes in the size or enhancement pattern of the hepatic nodules. Additional small DWI-hyperintense nodules were observed, and splenomegaly was present. Before T-DM1 treatment, the patient had no evidence of portal hypertension, thrombocytopenia, or splenomegaly (**Fig. 2A**). After T-DM1 initiation, thrombocytopenia and splenomegaly developed, and new hepatic nodules were detected at approximately the same time. Based on the temporal association with T-DM1 therapy, the accompanying thrombocytopenia and splenomegaly, and the atypical imaging findings, T-DM1-associated NRH was suspected. Liver biopsy was recommended for diagnostic confirmation; however, the patient declined the procedure. Therefore, the diagnosis of NRH remained unconfirmed.

**Fig. 3:**
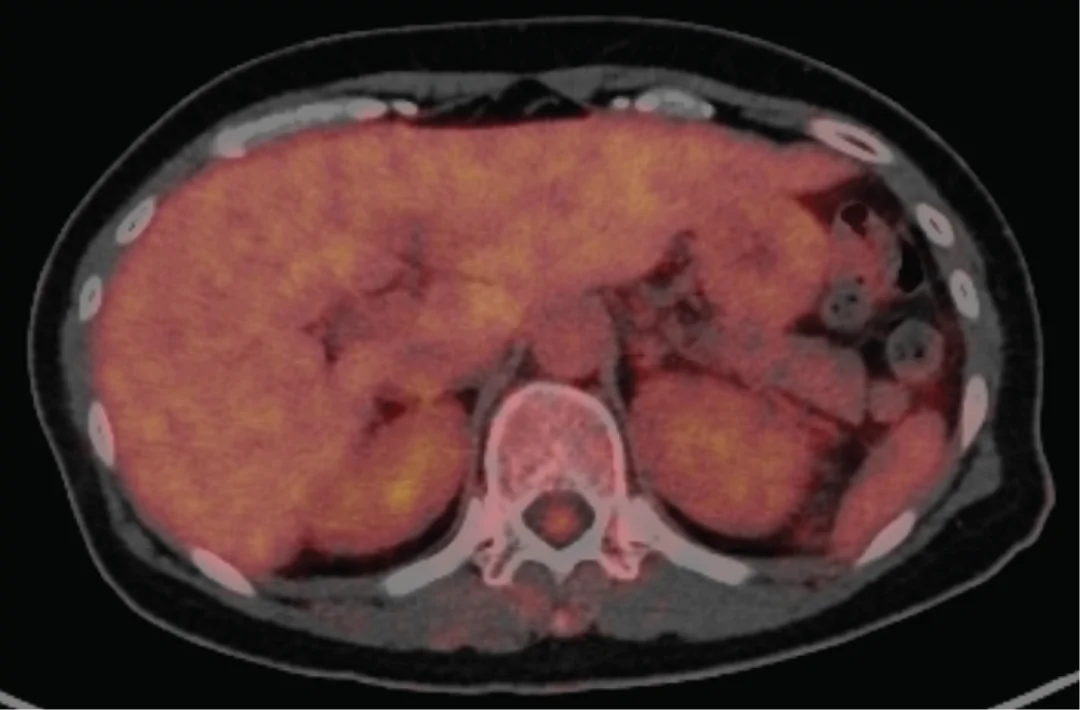
FDG-PET/CT findings after completion of T-DM1 therapy. No abnormal FDG uptake was observed in the hepatic lesions corresponding to the nodules detected on EOB-MRI, supporting the absence of metabolically active metastatic disease. EOB-MRI, gadolinium-ethoxybenzyl-diethylenetriamine pentaacetic acid-enhanced MRI; FDG, fluorodeoxyglucose; T-DM1, trastuzumab emtansine

During T-DM1 therapy, thrombocytopenia progressed to grade 2 according to the common terminology criteria for adverse events.^3)^ Although liver enzyme elevation and thrombocytopenia were observed, T-DM1 was continued with intermittent treatment interruptions, and all 14 planned cycles were ultimately completed. EOB-MRI performed after completion of T-DM1 showed no substantial changes in the hepatic nodules compared with those observed during treatment, and the lesions showed no abnormal fluorodeoxyglucose (FDG) uptake on PET/CT (**Fig. 3**). On subsequent follow-up EOB-MRI, the hepatic nodules showed no marked progression, although additional small DWI-hyperintense nodules remained visible (**Supplementary Fig. 5**). Non-contrast CT showed persistent splenomegaly (**Fig. 2B**).

One year after completion of T-DM1, the patient continued to receive an aromatase inhibitor and showed no evidence of breast cancer recurrence. Follow-up EOB-MRI showed no substantial changes in the hepatic lesions or findings suggestive of portal hypertension (**Supplementary Fig. 6**). The patient provided written informed consent for publication of this case report.

## DISCUSSION

Although adjuvant T-DM1 significantly reduces the risk of recurrence in patients with HER2-positive breast cancer,^1)^ some patients require dose reduction or treatment discontinuation due to hepatotoxicity and thrombocytopenia. In clinical trials, transaminase elevation has been reported as a relatively frequent adverse event. Grade 3 or 4 AST elevation occurred in 4.2% of patients in the EMILIA trial and in 2.0% of patients in the TH3RESA trial.^1,4)^ However, NRH associated with T-DM1 has been reported in recent years and has drawn attention as a clinically important form of liver injury. NRH was observed in 0.3% (2/740) of patients in the KATHERINE trial and in 0.7% (3/403) of patients in the TH3RESA trial.^2,4)^ Nevertheless, NRH is clinically important because it may lead to non-cirrhotic portal hypertension and may be difficult to distinguish from liver metastasis when newly detected hepatic nodules develop during T-DM1 therapy.

NRH is thought to result from heterogeneous intrahepatic blood flow, with atrophy developing in hypoperfused regions and compensatory hyperplasia occurring in relatively hyperperfused regions. The underlying mechanism is presumed to involve injury or obstruction affecting small portal venules or sinusoids. In drug-induced NRH, sinusoidal endothelial dysfunction or sinusoidal obstruction syndrome-like changes are thought to precede the development of NRH, potentially leading to non-cirrhotic portal hypertension.^5)^ The DM1 component of T-DM1 may cause sinusoidal endothelial injury and thereby contribute to the development of NRH. Furthermore, because T-DM1 is primarily metabolized and degraded in the liver and reticuloendothelial system, and is excreted mainly via the biliary and fecal routes, the occurrence of hepatic adverse events is pharmacokinetically plausible.^6)^

Previous reports of T-DM1-associated NRH are summarized in **Table 1**.^7–11)^ In 5 reports published between 2016 and 2020, 7 patients with metastatic or recurrent HER2-positive breast cancer developed T-DM1-associated NRH after prolonged exposure to T-DM1, ranging from 16 to 77 cycles. In these cases, NRH was generally suspected after manifestations of portal hypertension, particularly thrombocytopenia and splenomegaly. All previously reported cases were confirmed histopathologically by liver biopsy. In contrast, in the present case, NRH was suspected during adjuvant T-DM1 therapy after 9 cycles, when new hepatic nodules appeared concomitantly with thrombocytopenia and splenomegaly. The absence of these findings before T-DM1 initiation and their development during treatment support a temporal association between T-DM1 exposure and the suspected NRH. Although EOB-MRI findings are not specific for NRH, the hepatobiliary-phase findings were useful in the differential diagnosis. Differential diagnoses for the newly detected hepatic nodules included liver metastases, hepatocellular carcinoma, focal nodular hyperplasia, hemangioma, and other regenerative or hyperplastic nodules. Liver metastases usually appear as hypointense defects in the hepatobiliary phase because they lack functioning hepatocytes.^12)^ In contrast, regenerative or hyperplastic hepatocellular nodules may show iso- or hyperintensity, reflecting preserved hepatocellular function. A previous report of NRH described portal venous phase-predominant enhancement and a doughnut-shaped appearance in the hepatobiliary phase.^13)^ Similarly, the segment IV lesion in the present case showed a comparable enhancement pattern, with an appearance resembling the previously reported doughnut-shaped pattern (**Supplementary Fig. 6D** and **6F**). Therefore, the mildly hyperintense or isointense appearance of the lesions in the present case, together with thrombocytopenia, splenomegaly, and their temporal relationship with T-DM1 exposure, was considered compatible with a regenerative hepatic process and supported the noninvasive suspicion of T-DM1-associated NRH, although these findings were not diagnostic. Despite suspected NRH, T-DM1 was completed under close monitoring because the diagnosis remained unconfirmed. No radiologic progression of the hepatic lesions or marked worsening of liver-related findings was observed. When T-DM1-associated NRH is diagnosed or strongly suspected, treatment decisions should be individualized by balancing the oncological benefit of T-DM1 against the risk of progressive hepatic injury. The principal limitation of this report is the lack of histopathological confirmation because the patient declined liver biopsy. Therefore, neither the diagnosis of NRH nor a causal relationship with T-DM1 can be definitively established. Nevertheless, the temporal association with T-DM1 exposure, concurrent thrombocytopenia and splenomegaly, atypical imaging findings, absence of FDG uptake, and subsequent radiologic stability collectively supported the suspicion of T-DM1-associated NRH. Thus, when new hepatic nodules develop during T-DM1 therapy, NRH should be considered in the differential diagnosis, particularly when accompanied by findings suggestive of portal hypertension, while recognizing that histopathological examination is required for a definitive diagnosis.

**Table 1:** Previous reports of T-DM1-associated NRH in the literature

Study	Age (years)	Publication year	Number of T-DM1 cycles	Imaging finding/laboratory data	Diagnostic mode
Force et al.^7)^	66	2016	23	Ascites, esophageal varices, hepatic encephalopathy, portal vein thrombosis, and thrombocytopenia	Biopsy
Force et al.^7)^	50	2016	22	Esophageal varices and thrombocytopenia	Biopsy
Prochaska et al.^8)^	73	2016	21	Splenomegaly, esophageal varices, and thrombocytopenia	Biopsy
Lepelley et al.^9)^	48	2018	16	Splenomegaly	Biopsy
Benguerfi et al.^10)^	52	2019	23	Splenomegaly and thrombocytopenia	Biopsy
Benguerfi et al.^10)^	48	2019	77	Ascites and edema	Biopsy
Hassan et al.^11)^	61	2020	39	Mesenteric vein varices	Biopsy
Present case	58	2026	9	Splenomegaly and thrombocytopenia	Imaging findings

Five reports published between 2016 and 2020 described 7 cases in patients with metastatic or recurrent breast cancer treated with 16 to 77 cycles of T-DM1; portal hypertension-related findings were reported, and the diagnosis was confirmed by liver biopsy. The present case is unique as an adjuvant case in which T-DM1-associated NRH was suspected noninvasively, without biopsy, based on imaging findings and clinical course after completion of 9 cycles.

NRH, nodular regenerative hyperplasia; T-DM1, trastuzumab emtansine

## CONCLUSIONS

T-DM1-associated NRH was suspected based on noninvasive imaging and clinical findings, although histopathological confirmation was not obtained. When new hepatic nodules develop during adjuvant T-DM1 therapy, T-DM1-associated NRH should be considered in the differential diagnosis, while recognizing that liver biopsy is required for a definitive diagnosis.

## SUPPLEMENTARY MATERIALS

Supplementary Fig. 1 Initial mammographic finding. Mammography showed a spiculated mass in the upper outer quadrant of the right breast.

Supplementary Fig. 2 Breast ultrasonography findings. (**A**) A 3.2-cm hypoechoic mass was observed in the right breast. (**B**) A mildly enlarged axillary lymph node was observed.

Supplementary Fig. 3 Non-contrast abdominal MRI performed after nine cycles of T-DM1. (**A**) T2-weighted image showing mild hyperintensity of a lesion in liver segment IV. (**B**) Diffusion-weighted image showing hyperintensity of the corresponding lesion.

Supplementary Fig. 4 Abdominal ultrasonography findings of the hepatic lesions after nine cycles of T-DM1. (**A**) A slightly irregular 13-mm hypoechoic lesion in hepatic segment IV. (**B**) A 14-mm hypoechoic lesion in the anterior aspect of hepatic segments III/IV.

Supplementary Fig. 5 Follow-up EOB-MRI findings of hepatic nodular lesions in segments III and IV after completion of 14 cycles of T-DM1. (**A**) Arterial phase image showing faint hyperintensity. (**B**) Portal venous phase image showing the lesions most conspicuously. (**C**) Transitional phase image showing persistent hyperintensity. (**D**) Hepatobiliary phase image showing mild hyperintensity of the segment III lesion and approximately isointensity of the segment IV lesion, without definite defects. (**E**) Diffusion-weighted image showing hyperintensity of the lesions and additional small DWI-hyperintense nodules.

Supplementary Fig. 6 Follow-up EOB-MRI findings 1 year after completion of T-DM1. The hepatic nodular lesions showed no marked change compared with the previous EOB-MRI, while the segment III lesion had become less conspicuous. (**A**) Arterial phase image. (**B**) Portal venous phase image. (**C**) Transitional phase image. (**D**) Hepatobiliary phase image. (**E**) Diffusion-weighted image. (**F**) Coronal image in the hepatobiliary phase.

## DECLARATIONS

### Availability of data and materials

The datasets supporting the conclusions of this case report are included within the article. Additional de-identified clinical information and imaging data may be available from the corresponding author upon reasonable request, subject to institutional review and patient privacy/consent considerations.
