# Supplementary material for: Nodular Regenerative Hyperplasia Suggested by Noninvasive Findings during Adjuvant Trastuzumab Emtansine Therapy for Human Epidermal Growth Factor Receptor 2-Positive Breast Cancer
Source: Surg Case Rep. 2026 Sep 17;12(1):26-0344. doi: 10.70352/scrj.cr.26-0344 (PMC13601968; doi:10.70352/scrj.cr.26-0344)

Supplementary Figure

Supplementary Figure 1

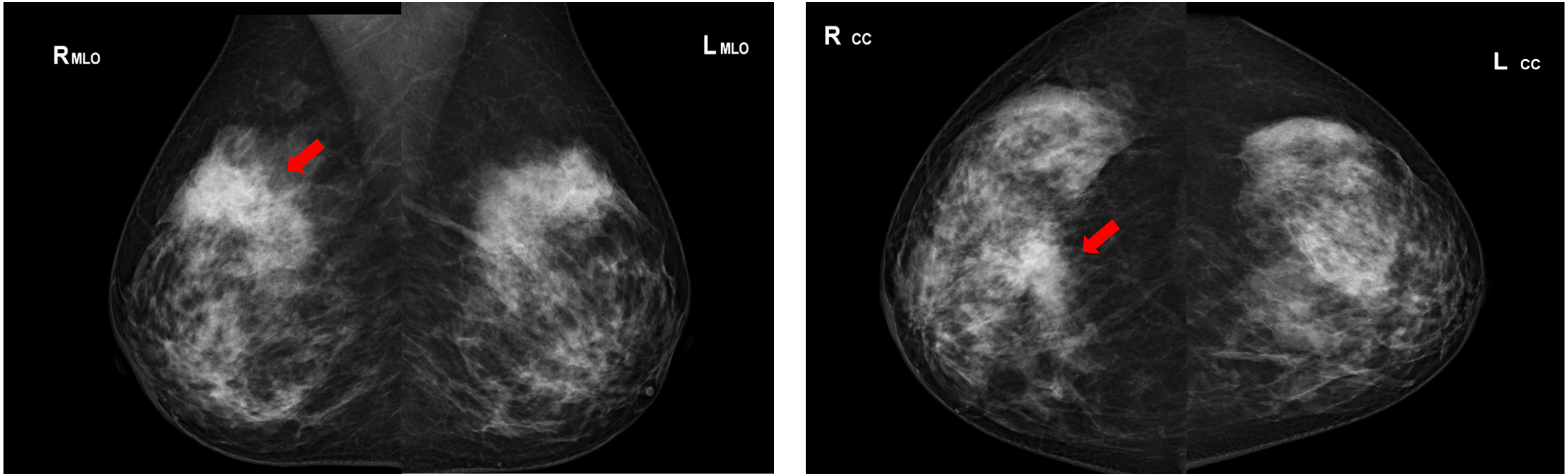

Supplementary Figure 2

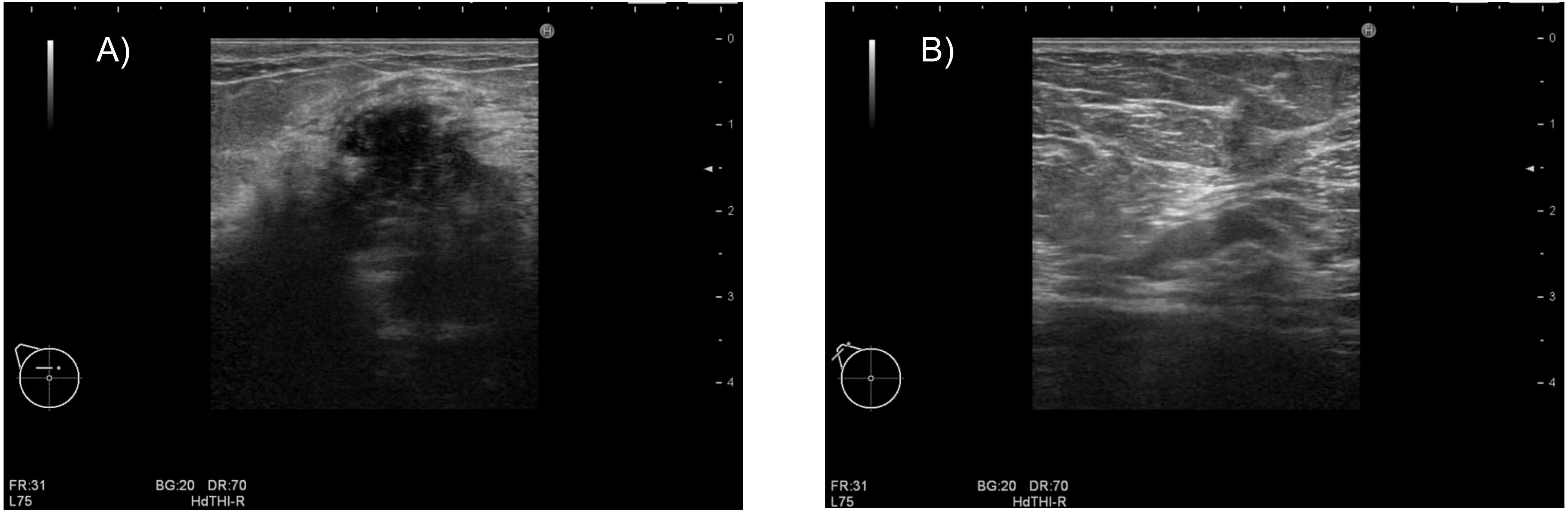

Supplementary Figure 3

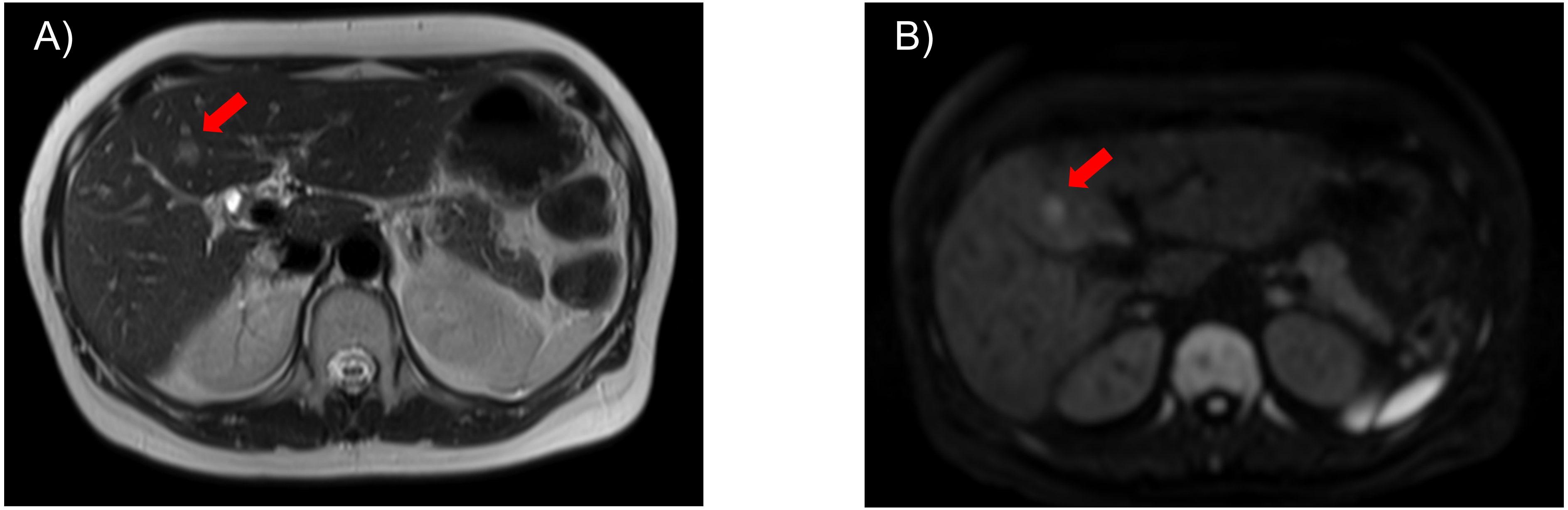

Supplementary Figure 4

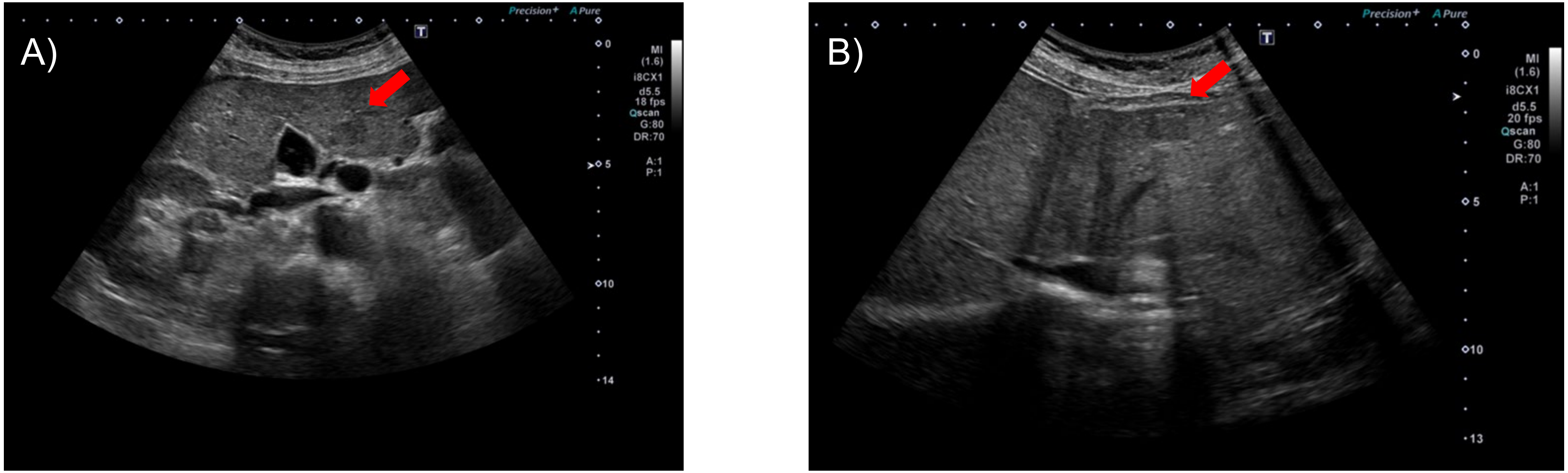

Supplementary Figure 5

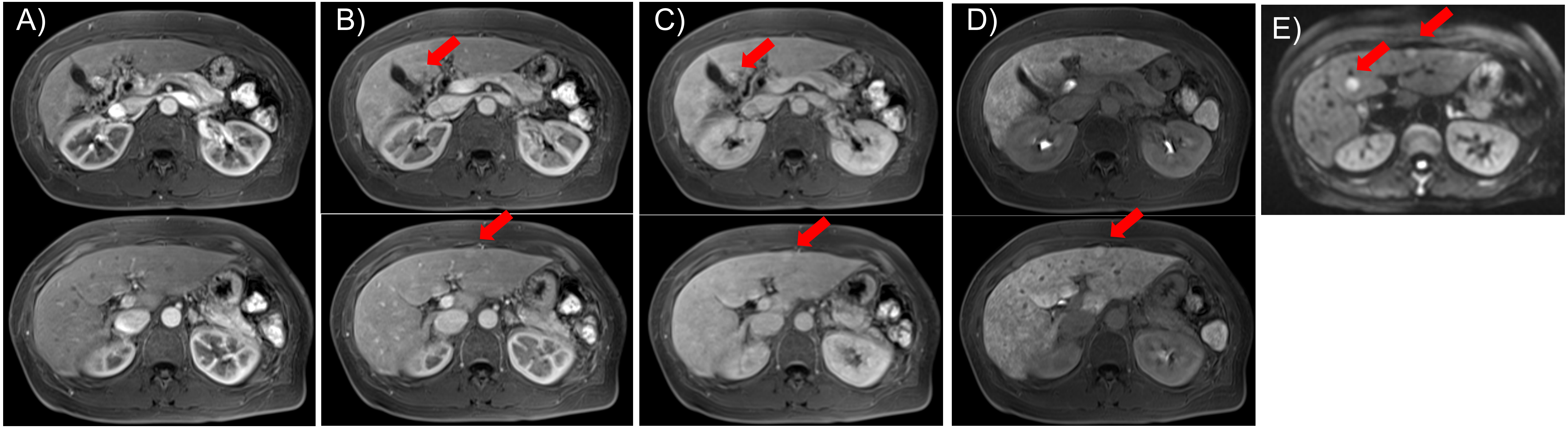

Supplementary Figure 6

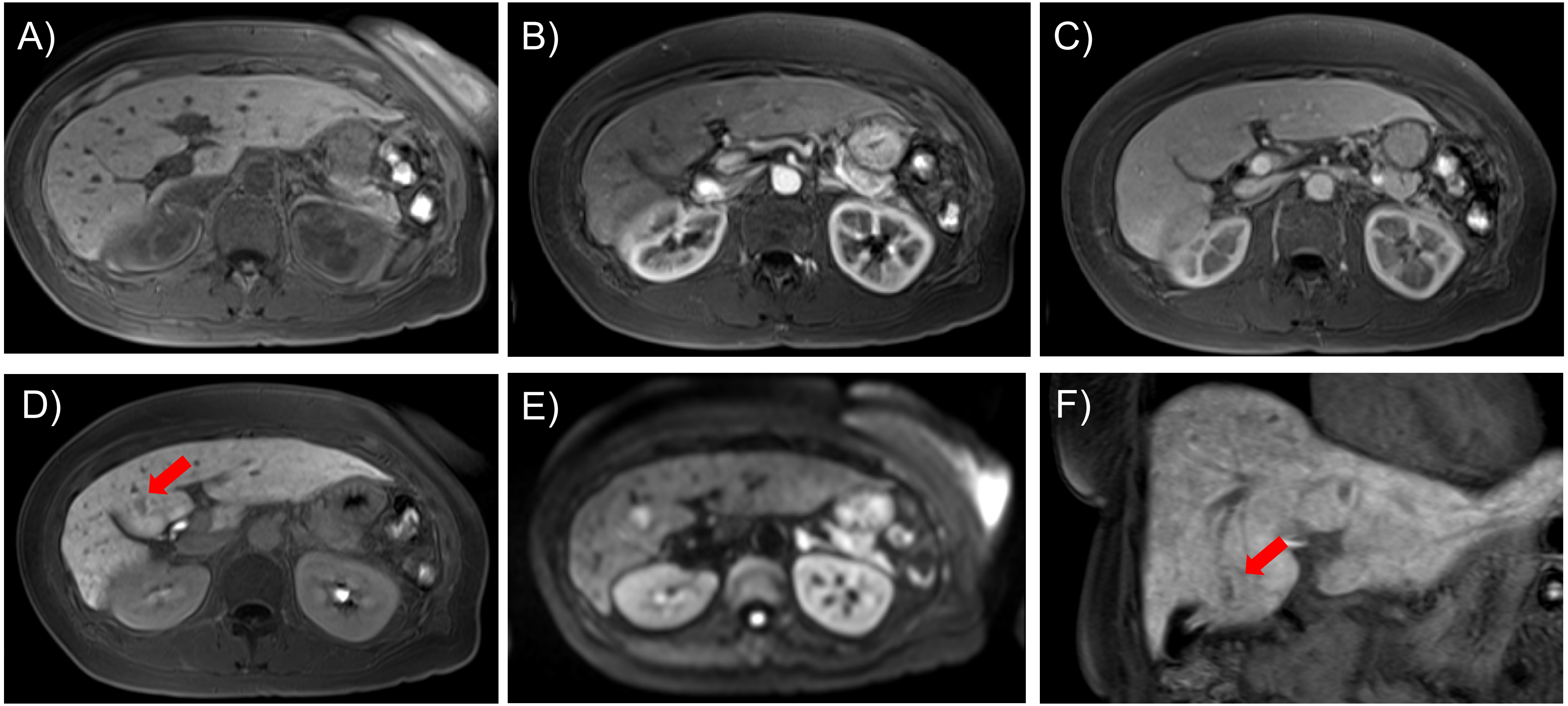

Supplement: Supplementary file 1 [file scr-12-01-26-0344-s001.pdf]
